# Incidence, Risk Factors, and Stroke Prevention During Transcatheter Aortic Valve Implantation

**DOI:** 10.31083/RCM26867

**Published:** 2025-04-22

**Authors:** Sannidhya Misra, Arif A Khokhar, Carla Lucarelli, Saud Khawaja, Ghada W Mikhail

**Affiliations:** ^1^Department of Cardiology, Hammersmith Hospital, Imperial College Healthcare NHS Trust, W12 0HS London, UK

**Keywords:** transcatheter aortic valve implantation, stroke, silent brain injury, cerebroembolic protection devices

## Abstract

Stroke remains a significant, potentially life-threatening complication following transcatheter aortic valve implantation (TAVI). Moreover, the rate of strokes, particularly disabling strokes, has not diminished over time despite improvements in pre-procedural planning and implantation techniques. The mechanisms of stroke in TAVI patients are complex, and identifying consistent risk factors is challenging due to evolving patient profiles, varied study cohorts, and continuous device modifications. Multiple pharmacological and mechanical treatment strategies have been developed to mitigate the risk of stroke, particularly as TAVI expands toward younger populations. This review article discusses the pertinent factors in the evolution of stroke post-TAVI, appraises the latest evidence and techniques designed to reduce the risk of stroke, and highlights future strategies and technologies to address this unmet need.

## 1. Introduction

Transcatheter aortic valve implantation (TAVI) has emerged as a safe and effective treatment strategy for severe aortic stenosis (AS). Over time, advancements in procedural techniques and valve design have expanded the applicability of TAVI across all surgical-risk categories for severe AS, with randomized control trials consistently demonstrating favorable outcomes for TAVI compared to surgical aortic valve replacement (SAVR), even in low-risk patients [1, 2]. However, despite the broadening demographic of patients eligible for TAVI, the rate of periprocedural stroke has remained relatively constant.

This review aimed to comprehensively understand the incidence, mechanisms, and key risk factors contributing to stroke after TAVI. It also discusses current preventative strategies, such as cerebroembolic protection (CEP) devices, and highlights the need for enhanced strategies and research to improve stroke prevention after TAVI.

## 2. Research Methodology

This review paper was constructed by systematically analyzing published literature on stroke in TAVI patients. This involved searching relevant databases (PubMed, EMBASE, and Cochrane Library) using specific keywords (e.g., “TAVI”, “stroke”, “DW-MRI”). Studies meeting the inclusion criteria (such as those focusing on stroke incidence, silent strokes, and neurological assessment) were selected. Data extracted from these studies included stroke rates, stroke types, and the influence of procedural factors. This information was then synthesized and analyzed to provide a comprehensive overview of stroke in the context of TAVI.

## 3. Definition of Stroke

Stroke is largely a clinical diagnosis further supported by cerebral imaging. Clinically, any new focal or global neurological deficit persisting for more than 24 hours is defined as a stroke. Studies evaluating stroke risk post-TAVI used a combination of these methods to diagnose stroke. In the initial studies, stroke could be reported by any physician and later confirmed by a specialist stroke or neurology doctor. More recently, studies have used routine imaging to help guide the diagnosis, as demonstrated in Table 1 (Ref. [1, 2, 3, 4, 5, 6, 7, 8, 9, 10, 11]).

**Table 1.** **Methodology used to report stroke risk in selected randomized controlled trials for TAVI procedures**.

Study	Year	Number of patients	Methodology for stroke assessment	Initial assessment	Reported stroke rate
TAVR vs SAVR Studies					
PARTNER B [3]	2011	348	Clinical	Any physician	6.7%
CoreValve High Risk [4]	2014	390	Clinical	Any physician	4.9%
Notion [5]	2015	145	Clinical	Any physician	1.4%
PARTNER 2 [6]	2016	1011	Clinical +/- MRI	Any physician	5.5%
SURTAVI [7]	2017	864	Clinical +/- MRI	Any physician	4.5%
PARTNER 3 [1]	2019	496	Clinical +/- MRI	Neurologist or stroke specialist	0.6%
Evolut Low Risk [2]	2019	725	Clinical and MRI	Neurologist or stroke specialist	3.0%
SCOPE I [8]	2020	372	Clinical and MRI	Neurologist or stroke specialist	2.0%
CEP Studies					
CEP (control)					
PROTECTED TAVR [9]	2020	3000	Clinical and MRI	Neurologist or stroke specialist	2.3% (2.9%)
CLEAN TAVI [10]	2017	363	Clinical and MRI	Neurologist or stroke specialist	5.6% (9.1%)
REFLECT II [11]	2020	220	Clinical and MRI	Neurologist or stroke specialist	8.3% (5.3%)

TAVI, transcatheter aortic valve implantation; SAVR, surgical aortic valve replacement; CEP, cerebroembolic protection; MRI, magnetic resonance imaging; TAVR, transcatheter aortic valve replacement.

Evidence of overt stroke appears relatively lower when compared to clinically silent stroke or silent brain infarcts (SBIs) post-TAVI. A meta-analysis of 39 studies reviewing patients post-TAVI with cerebral diffusion-weighted magnetic resonance imaging (DW-MRI) found that over 70% of patients had new vascular lesions in their head confirming a stroke; however, only 8% of these patients had any focal neurological deficit to identify a clinical stroke [12].

Silent brain infarcts are detected using neuroimaging techniques, primarily DW-MRI. This technique is sensitive to the subtle tissue changes produced by small strokes that may not cause noticeable symptoms. While DW-MRI is the gold standard for identifying SBIs, studying these events has limitations. Not all TAVI patients undergo preoperative cerebral imaging, making it difficult to determine if SBIs are present before the procedure. This lack of baseline data makes establishing a direct causal relationship between TAVI and SBIs challenging. Moreover, comparing SBI incidences in TAVI patients to the general population is difficult, as there is usually no reason to study SBIs in individuals who have not undergone a procedure. This limits our understanding of the extent to which TAVI contributes to SBI development. Thus, the long-term clinical significance of SBIs in TAVI patients remains an area of ongoing investigation, particularly as TAVI is increasingly performed on younger patients.

When studied post-cardiac or even after non-cardiac procedures, SBIs have been associated with post-procedural cognitive dysfunction in the acute or subacute phase. There is also evidence that this early cognitive dysfunction can progress to more long-term deficits and increased mortality [13, 14]. However, long-term studies analyzing SBIs in patients post-TAVI must quantify this impact, especially as TAVI progresses to younger cohorts.

A systematic analysis of 399,491 TAVI patients from randomized controlled trials (RCTs) (6370 patients), registries (392,288 patients), and CEP device studies using DW-MRI (833 patients) evaluated the incidence of stroke post-procedure [15]. The incidence of ischemic cerebrovascular events 30 days after TAVI was significantly higher (6.36%) in RCTs focusing on CEP devices compared to non-CEP device-related RCTs (3.86%) or registries (2.29%) [15].

RCTs have been found to under-report or provide incomplete data, which may be the underlying cause of the lower incidence reported in self-reported documentation. RCTs that focused on CEP devices reported a higher incidence. This is likely due to the use of DW-MRI, which detects clinical and subclinical stroke [16]. Indeed, studies using DW-MRI have shown that 60% to 90% of patients develop new silent cerebral lesions after TAVI, regardless of the vascular access route or device type used [17, 18, 19].

A greater occurrence of strokes was also reported in patients who had a standardized neurological assessment post-TAVI compared to studies that did not utilize a neurological check-up (4.03% with check-up vs. 2.47% without), particularly for non-compromising strokes (2.29% with check-up vs. 0.77% without). However, the one-year mortality rate was lower in the groups with a scheduled neurological follow-up than in cases lacking a scheduled neurological evaluation [15].

## 4. Incidence and Clinical Relevance 

Three distinct phases of stroke risk following TAVI have been identified: The immediate periprocedural period (within 72 hours), the early phase (up to 30 days), and the delayed phase (beyond 30 days). The heightened stroke risk is most prominent during the immediate periprocedural and early phases, while the long-term or delayed stroke risk appears to be more closely related to pre-existing comorbidities rather than the TAVI procedure itself [20].

The Society of Thoracic Surgeons and the American College of Cardiology registry followed 101,430 patients who underwent TAVI treatment from 2011 to 2017 [21]. This registry reported a 2.3% incidence of stroke within 30 days, while transient ischemic attacks (TIAs) were reported at a rate of 0.3%. Meanwhile, there was no observed decrease in the occurrence of stroke over the years, suggesting that advancements in device technology or procedural technique did not lead to a significant reduction in cerebral embolic events. In more recent studies, such as the Evolut low-risk or PARTNER 3 trials, the reported stroke rate at 30 days post-procedure was 0.5–0.6%, even in patients classified as low-risk [1, 2]. Further studies indicate an increased risk of stroke within the first year post-TAVI, suggesting that patients are susceptible to both immediate neurological deficits and longer-term cognitive impairments due to ongoing risk factors as well as silent cerebral infarcts [22, 23, 24].

Notably, the impact of stroke extends beyond the immediate neurological deficit, with a significant percentage of stroke patients facing challenges such as limitations in social and recreational activities, neurocognitive impairments, and the need for additional support following a stroke after TAVI. Moreover, the occurrence of stroke was linked to a notable sixfold increase in the risk of mortality within 30 days [21].

## 5. Pathophysiology of Acute Peri-Procedural Stroke

Most cerebrovascular events after TAVI are related to an ischemic source, with the majority attributed to an embolic source [25, 26]. The nature of these emboli is varied, as are the theories underlying their source and contribution to stroke risk. In a study evaluating the incidence and histopathology of debris collected by CEP devices, debris was collected in 85% of cases. Subsequently, 74% of these were thrombotic or fibrin material, 63% were tissue-derived debris, and 17% were found to have amorphous calcified material [27]. This also supports the idea that the stroke mechanism in the acute period is most likely due to the embolization of debris, specifically calcium, tissue, thrombus, or atheroma.

**Fig. 1.**
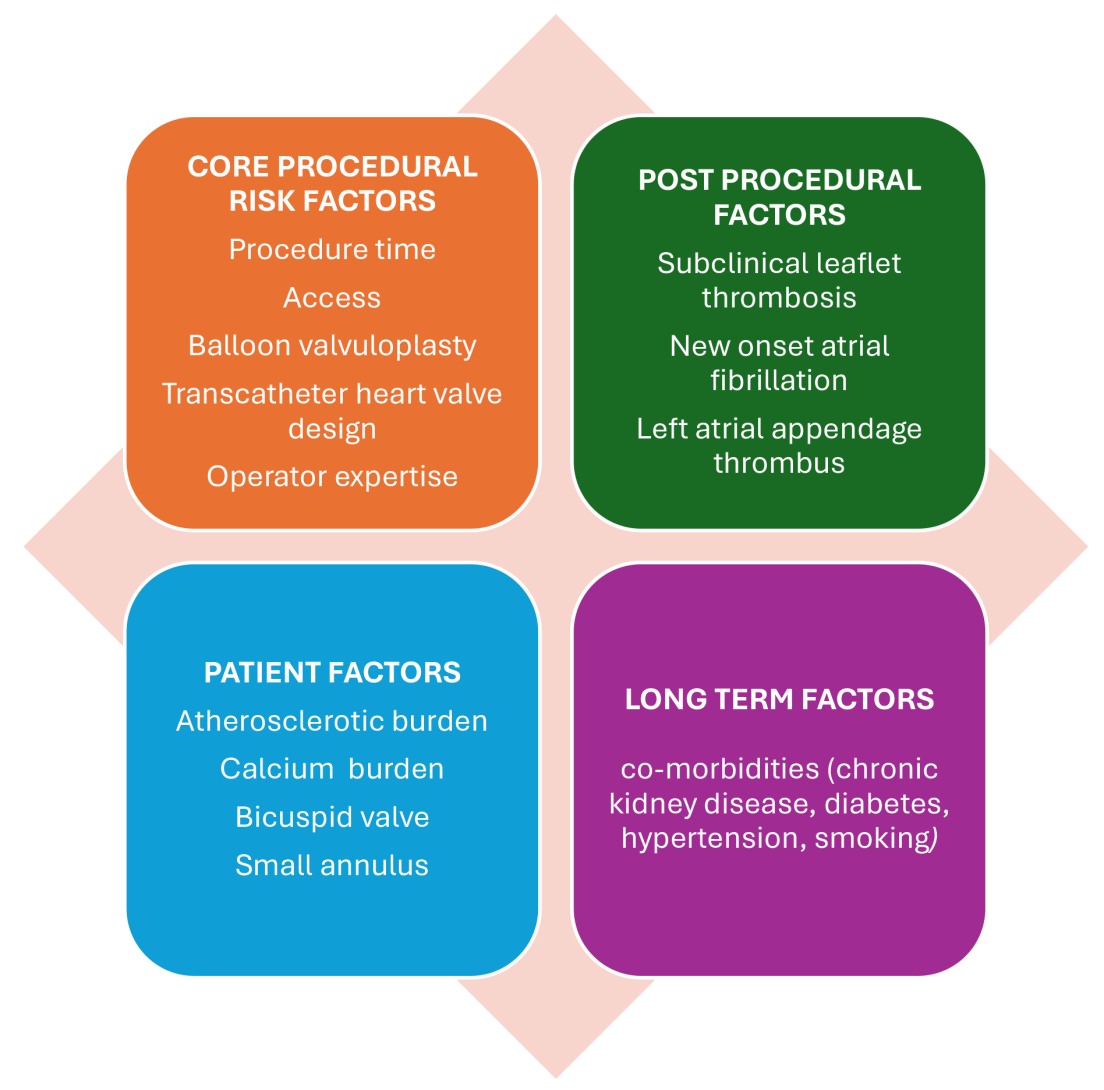
**Risk factors for stroke post-transcatheter aortic valve implantation (TAVI)**.

TAVI patients are often complex, with varied demographics and a large mix of baseline risk factors and co-morbidities. Multiple studies [25, 26, 28] have been conducted to predict the risk factors for stroke, and these continue to provide inconsistent responses, largely due to the varying cohorts and exponential growth in device and equipment options. Therefore, the risk factors are likely to overlap, and individualized risk assessments play an important role in predicting stroke risk after TAVI. Fig. 1 provides an overview of these risk factors.

## 6. Procedural Risk Factors 

### 6.1 Procedure Time 

Multiple factors may contribute to increased time in the catheterization lab, including increased debris dislodgement following wire manipulation, valve dilatation or valve repositioning, and alternate access routes. These factors can prolong a procedure and simultaneously increase the risk of stroke [28].

Additionally, manipulating vessels can damage endothelial tissue and activate the coagulation cascade. This, alongside the prothrombotic equipment used during the procedure, increases the risk of thrombus formation and embolization. Unfractionated heparin is the intraprocedural antithrombotic therapy of choice for TAVI patients. The ease of monitoring with activated clotting time (ACT) and the ability to reverse protamine allow for a safe balance of clotting and bleeding risk [29].

However, optimal ACT management remains an area of debate. While there is no universally accepted target ACT at the end of the procedure, most centers aim for an ACT over 250 seconds. Protamine use varies by center and is often guided by the operator’s assessment of bleeding risk versus the need for rapid heparin reversal. Some institutions may routinely administer protamine to all patients, while others reserve it for cases with prolonged ACT or those at high risk of bleeding.

Interestingly, recent studies have investigated the relationship between heparin antagonism with protamine and stroke incidence. However, while some studies suggest a potential association between protamine use and increased stroke risk, others have found no significant correlation. Thus, further research is needed to clarify this relationship and determine the optimal strategy for ACT management and protamine use in TAVI patients [30, 31].

### 6.2 Alternative Access

TAVI is conventionally performed through transfemoral access, but alternate routes are used in patients with peripheral arterial disease or hostile iliofemoral access. A 2023 registry of 1707 patients undergoing TAVI via the transfemoral (30.3%), transaxillary (32%), or transaortic access (37.6%) reported a higher rate of stroke/TIA in non-femoral access routes [32]. Another study compared transcaval and transaxillary access for TAVI across eight experienced centers using data from the Society of Thoracic Surgeons-American College of Cardiology Transcatheter Valve Therapy (STS/ACC TVT) Registry (2017–2020). Among 238 transcaval and 106 transaxillary procedures, stroke, and transient ischemic attacks were five times less common with transcaval access (2.5% vs. 13.2%). Meanwhile, both non-femoral approaches had more complications than transfemoral access (1.7%), but transcaval TAVI showed lower stroke risk and comparable bleeding risk [33]. Transapical TAVI had a lower 30-day stroke/TIA risk (2.7%) than retrograde transarterial implantation of the same Edwards SAPIEN valve (4.2%). Similarly, this finding is likely due to the minimal catheter manipulation required in the ascending aorta and arch via the transapical method, reducing the chance of dislodging atheromatous plaques [34].

### 6.3 Transcatheter Heart Valve Design

Initial studies on using self-expandable valves in TAVI reported a slightly higher risk of stroke, likely due to their larger size and potential for more extensive manipulation during positioning. However, a more recent meta-analysis that compared the 30-day incidence of stroke following TAVI using self-expandable versus balloon-expandable valve prostheses found no significant difference in stroke rates between the two types of valve prostheses within the first 30 days post-implantation. This suggests that both self-expandable and balloon-expandable valves are similarly safe concerning stroke risk in the short-term period after TAVI, providing clinicians with flexibility in choosing the appropriate valve type based on other technical factors [35].

### 6.4 Adjunctive Procedures

Balloon aortic valvuloplasty (BAV) is used pre- or post-valve deployment in TAVI procedures to optimize valve implantation. However, this additional manipulation of the diseased aortic valve poses a theoretical risk of more debris dislodging. Nonetheless, current studies investigating the incidence of new lesions on cerebral DW-MRI found no difference between pre-BAV + TAVI versus the direct TAVI approach [36, 37, 38].

Leaflet modification procedures such as bioprosthetic or native aortic scallop intentional laceration to prevent iatrogenic coronary artery obstruction (BASILICA) have been found in early registries to be associated with a significant stroke risk, up to 10% at 30 days, with no additional increase noted at the 1-year follow-up. This elevated stroke risk can be attributed to the dislodgement of calcific material during leaflet laceration; meanwhile, further technical and device modifications are expected to address this [39].

## 7. Patient-Related Risk Factors

### 7.1 Bicuspid Valves

Bicuspid aortic valves are the most common cardiac congenital anomaly [40, 41]. Procedural difficulties in performing TAVI in bicuspid valves are well recognized but have significantly improved after the technique refinement technique and valve design [42]. However, the increased calcium burden and the need for balloon valvuloplasty or valve repositioning continue to enhance the overall risk of stroke by enhancing the procedural risk factors [43]. Thrombus formation on valve leaflets is also reported to be higher in bicuspid aortic valves [44].

A registry-based prospective cohort study of patients undergoing TAVI analyzed 2691 propensity score-matched pairs of bicuspid and tricuspid aortic stenosis. The 30-day stroke rate was significantly higher for bicuspid vs. tricuspid aortic stenosis (2.5% vs. 1.6%; HR, 1.57 [95% CI: 1.06 to 2.33]). However, the all-cause mortality was not significantly different between patients with bicuspid and tricuspid aortic stenosis at 30 days or 1 year [45]. A further cohort study reviewing low-risk patients undergoing TAVI reported no significant difference in stroke rates between bicuspid versus tricuspid aortic valves at 30 days (1.4% vs. 1.2%; HR, 1.14 [95% CI: 0.73 to 1.78]; *p* = 0.55) or 1 year (2.0% vs. 2.1%; HR 1.03 [95% CI: 0.69 to 1.53]; *p* = 0.89) [46].

### 7.2 Calcium Burden

**Fig. 2.**
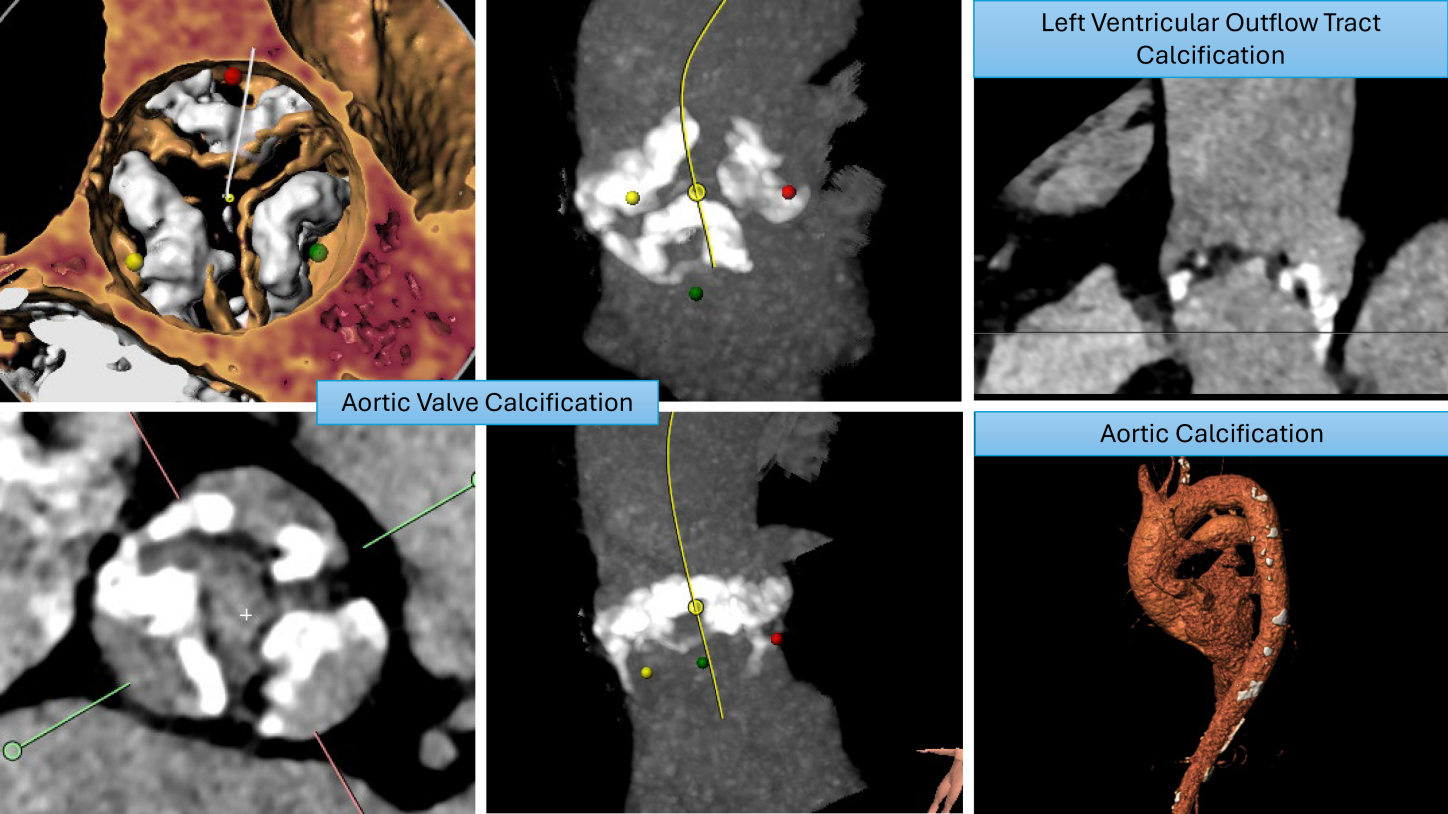
**Patterns of calcification that increase stroke risk**.

Increased aortic valve calcification increases the risk of acute stroke peri-procedurally due to the increased debris generated during the procedure. Pollari *et al*. [47], in a retrospective study analyzing computed tomography (CT) scans pre-TAVI procedure, reported a significantly increased risk of stroke associated with left ventricular outflow tract calcification. This was further evidenced by Maier *et al*. [48] in a retrospective study investigating risk factors for stroke post-TAVI, who reported a higher calcium volume, specifically in the left ventricular outflow tract (LVOT) and right coronary cusp (RCC), which was associated with higher stroke rates. Examples of significant aortic root calcification, which may increase stroke risk during TAVI, are illustrated in Fig. 2.

### 7.3 Atherosclerotic Burden 

Similar to the total calcium volume, atherosclerotic burden, particularly in the aortic arch and supra-aortic vessels, may also contribute to an elevated stroke risk during TAVI. Increased aortic arch atheroma was related to an increased risk of cerebrovascular events [49]. The anatomical location of the ascending aorta and aortic arch often means that large or mobile plaques in these regions will likely dislodge debris and embolism toward the cerebral circulation [50, 51].

## 8. Pathophysiology of Early Stroke (<30 Days) Post-Transcatheter Aortic Valve Replacement (TAVR) 

### 8.1 Atrial Fibrillation

Atrial fibrillation has been associated with higher stroke and mortality rates post-TAVI [52]. A study assessing arrhythmia incidence and burden post-TAVI used an implantable cardiac device inserted before TAVI and followed patients up for a minimum of 12 months. New onset atrial fibrillation (NOAF) was diagnosed in 19% of these patients, with a median onset time of 57 days. Additionally, 24% of patients had pre-existing AF (largely paroxysmal) with a median time of first AF recording post TAVI of 6 days, with no overall increase in AF burden [53]. Given the large number of NOAF cases, routine monitoring and timely initiation of treatment may provide favorable outcomes, but further research is required to confirm this.

### 8.2 Anticoagulation

The GALILEO trial, which investigated rivaroxaban use in patients post-TAVI without an established clinical indication for anticoagulation, was stopped before completion due to safety concerns. After an average follow-up of 17 months, data reported a higher risk of bleeding, thromboembolic events, and death in the rivaroxaban arm when compared to single antiplatelet therapy in the form of aspirin [54].

The 2022 ATLANTIS trial involved 1500 patients randomly allocated to receive either the oral anticoagulant apixaban or standard care, which included vitamin K antagonists or antiplatelet therapy, based on individual indications. The primary endpoints measured included the risk of stroke, mortality, and major bleeding. The results indicated no significant difference between the two groups regarding stroke risk or overall mortality. This finding suggests that apixaban offers no clear advantage over traditional treatments for patients requiring anticoagulation therapy, again highlighting the need for tailored approaches based on patient-specific factors [55].

### 8.3 Subclinical Leaflet Thrombosis

Subclinical leaflet thrombosis (SLT) is characterized by hypo-attenuated leaflet thickening (HALT) on imaging and may be associated with an increased risk of stroke [56].

In a systematic review of 11,098 patients, the incidence of SLT was 6% at a follow-up of 30 days. After a longer-term follow-up of patients with SLT, a 2.6-fold increase in the risk of stroke or TIA was found compared to patients without SLT (relative risk (RR): 2.56; 95% CI: 1.60 to 4.09; *p* < 0.00001) [57]. SLT in patients on oral anticoagulants had a relative risk reduction of 58% compared with those on antiplatelets (RR: 0.42; 95% CI: 0.29 to 0.61; *p* < 0.00001). Additionally, there was no difference in the risk for SLT between single and dual antiplatelet therapy (RR: 0.97; 95% CI: 0.72 to 1.29; *p* = 0.83).

A further smaller meta-analysis also supported the use of oral anticoagulation to reduce the risk of SLT (incidence rate ratio (IRR) 7.51, 95% CI: 3.24 to 17.37, I^2^ 62%, 95% CI: 0 to 87; *p* < 0.001). However, this study did not show an association between SLT and stroke risk (IRR 1.05, 95% CI: 0.32 to 3.47; *p* = 0.93) [58]. In the ADAPT-TAVR trial, where 229 patients were undergoing TAVI with no primary anticoagulation indication, edoxaban was found to halve the risk of SLT at 6 months, while the risk of stroke, TIA, and mortality was equivalent in the two groups [59].

Given the scarcity of long-term follow-up results to confirm the benefit of anticoagulation in preventing SLT, clinicians prefer a single antiplatelet regimen with aspirin or clopidogrel as the primary antithrombotic therapy unless there is a separate primary indication for anticoagulation.

### 8.4 Left Atrial Appendage Thrombus

The left atrial appendage represents an alternative source of cardioembolic stroke. A retrospective study evaluating the incidence of left atrial appendage thrombus (LAAT) via cardiac CT reported an 11% incidence of LAAT in the overall cohort being considered for TAVI and a 32% incidence in patients with pre-existing atrial fibrillation. Most of these patients also underwent transesophageal echocardiogram, which confirmed the CT findings on each occasion. The in-hospital stroke rate for patients who underwent TAVI with LAAT was found to be 20% compared to patients without LAAT (3.8%) [60].

Medical therapy with an anticoagulant represents the current management of AF and LAAT [61]. Left atrial appendage occlusion (LAAO) devices are increasingly used for patients with non-valvular AF, high bleeding risk, or other medical therapy intolerances [62].

The recent WATCH-TAVR study combined TAVI and an LAAO device to review any benefit in decreasing the thromboembolic and bleeding risk of patients with severe AS and AF. Patients were randomized into either a TAVI + LAAO arm with warfarin and aspirin for 45 days, followed by dual antiplatelet therapy until 6 months, or a medical therapy arm that received long-term anticoagulation or antiplatelets, depending on the clinician’s preference. The primary endpoint was all-cause mortality and incidence of stroke [63]. After 24 months of follow-up, TAVI and LAAO were non-inferior to TAVI and medical therapy (33.9% vs. 37.2%, HR: 0.86, 95% CI: 0.60 to 1.22; *p* < 0.001). The TAVI and LAAO groups reported an increased incidence of venous and arterial thrombus post-procedure and an increased intraprocedural time and contrast load. While these data suggest a potential benefit in specific patients, further clinical data and longer-term follow-up are required to determine the safety and efficacy of concomitant procedures fully.

## 9. Pathophysiology of Delayed Stroke (>30 Days) Post TAVR 

Data on the late risk of stroke post-TAVI are limited. Most research is focused on the first 30 days post-procedure, while some follow the patients for 12 months. A Danish study focused on predicting stroke risk factors in the early (within 30 days) and late (90 days to 5 years) phases post-TAVI. This study matched TAVI patients with control patients who possessed similar risk profiles to determine the risk of stroke post-procedure. It reported that TAVI was associated with a higher ischemic risk in the early phase, but the rate of stroke returned to expected rates by 1 year based on the patient’s co-morbidities [20].

Patient co-morbidities such as peripheral artery disease and previous stroke, which likely represent a high atheroma burden, were key factors in stroke risk prediction in the late phase [64]. Other known risk factors for stroke, such as age, female gender, hypertension, diabetes, chronic kidney disease, and heart failure, continue to play an important role, meaning strict monitoring and control of these factors in the long term should be imperative in managing stroke risk [65].

## 10. Alternate Theories for Stroke Mechanism

### 10.1 Air Embolism

Given that CEP devices have not fully reduced or eliminated the burden of post-TAVI stroke, alternative pathophysiological processes for peri-procedural stroke should be considered. In other cardiac procedures, such as thoracic endovascular aortic repair (TEVAR), air embolism has been reported as a likely pathway for stroke [66].

Makaloski *et al*. [67] used an aortic flow model to detect air bubbles in the supra-aortic vessels during thoracic stent-graft deployment, reporting mean volumes of 0.82 ± 0.23 mL to 0.94 ± 0.28 mL for the air released during the process. TAVI and TEVAR devices are manufactured similarly and prepared using similar saline flushing techniques.

INTERCEPTavi (NCT 05146037) is a novel first-in-human pilot RCT that demonstrates the neuroprotective benefits of minimizing air emboli by flushing TAVI valves with CO_2_ and saline (TAVI-CO_2_) versus standard saline only (TAVI-S). A brain MRI post-TAVI showed a significant reduction in the number of new cerebral lesions in TAVI-CO_2_ compared to the TAVI-S procedure, with nearly half the number of infarcts. A larger multi-center study is planned to confirm the neuroprotective benefits of minimizing air emboli; however, multiple large CEP trials have failed to provide convincing evidence regarding the neuroprotective benefits of only targeting solid emboli [68].

### 10.2 Hypoperfusion

Cerebral hypoperfusion is another possible cause of ischemic cerebrovascular disease. Rapid ventricular pacing, which is used in most TAVI procedures, can impair cerebral perfusion, but usually only for a short period. In patients with poor cardiac output, the effect of rapid pacing can be prolonged, and this extended period of hypoperfusion can further lead to ischemic stroke [69].

Similarly, significant periods of systemic hypotension due to procedural complications such as aortic regurgitation, heart block, or bleeding can also cause decreased perfusion to brain tissue and result in ischemic damage despite inotropic support. Hence, careful monitoring of patients and rapid management of these complications is essential to avoid long-term neurological damage [70].

## 11. Cerebroembolic Protection Devices

CEP devices were developed to reduce the risk of peri-procedural stroke during TAVI by filtering or deflecting debris bound for the cerebral circulation [71, 72]. Their mechanisms can be largely divided into two categories:

1—Deflection: This technique aims to guide debris away from the cerebral circulation, usually by restricting its path and redirecting it elsewhere.

2—Filter: This technique aims to capture debris before it reaches the brain.

Table 2 (Ref. [15, 16, 17, 18, 19, 25]) summarizes the main CEP devices currently available for clinical use and those being researched.

**Table 2.** **Summary of the characteristics of cerebroembolic protection devices [15, 16, 17, 18, 19, 25]**.

Device	Mechanism	Access	Trials	Summary of effect
Sentinel	Filter device	Radial	SENTINEL trial, PROTECTED TAVR, Clean TAVI, BHF PROTECT-TAVI	Captures 90–100% debris
	Does not cover vertebral artery			Inconclusive evidence around risk of stroke.
Triguard 3	Filter and deflection device	Femoral	REFLECT II Trial, ongoing studies	Increased bleeding and vascular complication, incomplete arch coverage in 40% cases
				No significant reduction in stroke or 30-day mortality
Emblok	Dual filter system	Femoral	EMBLOCK trial, ongoing studies	Effective in capturing debris
	Full Arch coverage			
ProtEmbo	Deflection device	Radial	PROTEMBO C trial, ongoing studies	Complete coverage in 98.2% patients
	Full Arch coverage			No safety concerns
Emboliner	Filter device	Femoral	Various studies in progress	No safety concerns
	Total Body coverage			

**Fig. 3.**
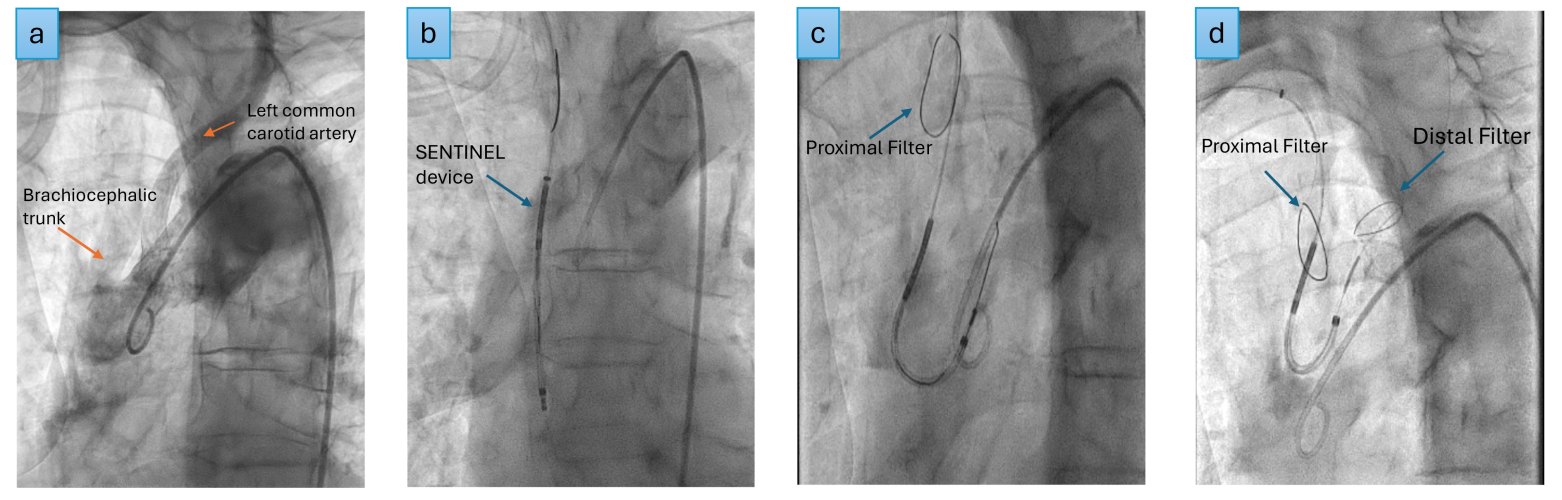
**SENTINEL deployment**. (a) Aortogram, (b) SENTINEL device inserted over the guide wire into the ascending aorta, (c) proximal filter deployed in the brachiocephalic trunk, (d) distal filter deployed in the left common carotid artery.

The SENTINEL (Boston Scientific, USA) remains the most commonly used and widely investigated CEP device (Fig. 3).

The PROTECTED TAVR represents an RCT comparing 1501 patients with SENTINEL to 1499 patients without the device, finding no significant difference in the incidence of stroke within 72 hours (2.3% CEP vs. 2.8% control, 95% CI: –1.7 to 0.5) or in the risk of mortality [73]. However, debilitating strokes occurred in fewer patients in the CEP group than in the control group (0.5% CEP vs. 1.3% in the control group, 95% CI: –1.5 to –0.1). This may be attributed to the device capturing larger particles while smaller particles continue to escape the device, causing non-disabling strokes [9].

The CLEAN TAVI RCT, which used DW-MRI, also confirmed no significant reduction in stroke rates in the SENTINEL device arm compared to the control arm. Instead, it revealed equivalent lesion distribution on MRI; however, the lesion volume was lower in the CEP group [10]. The clinical relevance of a lower lesion volume is unclear.

The BHF PROTECT-TAVI, a similar RCT to PROTECTED TAVR that used the SENTINEL device, aimed to study just under 8000 patients with a primary endpoint of all-cause stroke 72 hours post-TAVI procedure or by discharge (whichever occurs sooner); these data should be published in 2025. A further meta-analysis of PROTECTED TAVR and BHF PROTECT-TAVI is also planned, which should provide more substantial evidence regarding the use of CEP devices and their role in stroke prevention [74].

## 12. Future Perspectives

The significant sixfold increase in the 30-day mortality risk associated with stroke events post-TAVI presents a compelling argument for refining patient assessment protocols pre-TAVI. Robust assessment tools that consider both anatomical factors and patient history are requisite in accurately stratifying stroke risk. Furthermore, interdisciplinary cooperation in evaluating and implementing anticoagulation strategies for patients with new-onset atrial fibrillation and other thromboembolic risks remains vital.

While current antithrombotic therapies, including antiplatelet agents and oral anticoagulants, are commonly used, their effectiveness in reducing stroke risk remains an area of ongoing research. Indeed, the inconsistent findings from various studies regarding the impact of anticoagulation on subclinical leaflet thrombosis and stroke risk underscore the need for more targeted and long-term investigations. They also emphasize the need for comprehensive patient monitoring and individualized treatment plans.

The role of CEP devices remains crucial yet controversial. While CEP devices such as the SENTINEL aim to capture or deflect debris and theoretically reduce stroke risk, clinical trials such as PROTECTED TAVR have shown mixed results. Therefore, despite their theoretical benefits, the lack of significant reduction in stroke rates with CEP devices suggests their impact on clinical outcomes may be limited. This calls for further investigation into the efficacy of these devices and the development of improved technologies. An upcoming meta-analysis of the PROTECTED TAVR and BHF PROTECT-TAVI trials is expected to provide more robust evidence regarding the efficacy of CEP devices in preventing stroke. Alternate causes of stroke post-TAVI, such as air embolism, are in their initial phases of research, and larger clinical trials will reveal key evidence to guide this theory further.

A notable aspect of stroke risk post-TAVI is the occurrence of silent cerebral infarcts, which are detected more frequently than overt strokes. These silent infarcts, although not immediately symptomatic, are linked to long-term cognitive decline and potentially increased mortality. The gap in correlating silent infarct incidence with clinical outcomes points to an urgent need for longitudinal studies to elucidate the behavioral and cognitive health trajectory of patients post-TAVI and develop strategies to mitigate these risks.

## 13. Conclusions 

While TAVI represents a significant advancement in the treatment of severe aortic stenosis, the risk of stroke remains a critical issue. The data discussed in the paper reveal a complex interplay of factors contributing to stroke risk post-TAVI, underscoring both the progress made and the significant challenges that remain. Addressing this challenge will require a multifaceted approach, incorporating technological innovation, improved procedural techniques, and personalized patient care. Moreover, continued research and development in these areas are essential to enhancing the safety and outcomes of TAVI procedures.
